# Chemically Modified Hyaluronic Acid for Prevention of Post-Surgical Adhesions: New Aspects of Gel Barriers Physical Profiles

**DOI:** 10.3390/jcm11040931

**Published:** 2022-02-11

**Authors:** Luz Angela Torres-de la Roche, Véronique Bérard, Maya Sophie de Wilde, Rajesh Devassy, Markus Wallwiener, Rudy Leon De Wilde

**Affiliations:** 1 University Hospital for Gynecology, Pius Hospital, University Medicine Oldenburg, 26121 Oldenburg, Germany; de.wilde@yahoo.com (M.S.d.W.); rajeshdevassy@gmail.com (R.D.); rudy-leon.dewilde@pius-hospital.de (R.L.D.W.); 2 Laboratoire Interdisciplinaire Carnot de Bourgogne, UFR des Sciences de Santé, 21000 Dijon, France; veronique.berard@u-bourgogne.fr; 3 University Hospital for Obstetrics and Gynecology, University Medicine Heidelberg, 69120 Heidelberg, Germany; markus.wallwiener@gmail.com

**Keywords:** hyaluronic acid, crosslinked polymer, auto-crosslinked polymer, peel strength, viscosity, elasticity, extrusion force, adhesion prevention

## Abstract

This study was conducted to provide information regarding the chemistry—including structure, synthesis, formulation, and mechanical properties—of two types of chemically modified anti-adhesion gels made of hyaluronic acid. Gel A (Hyalobarrier^®^) and gels B and C (HyaRegen^®^ and MetaRegen^®^) that are used in postsurgical adhesion prevention. To date, little information is available on their physicochemical attributes. This information is necessary in order to understand the differences in their in vivo behavior. Methods: Comparative analyses were conducted under laboratory-controlled conditions, including measuring the shear viscosity, storage modulus G’, peel strength, and extrusion forces. Results: All polymers exhibited viscoelastic behavior. Polymer A showed a shear viscosity approximately three times larger than both polymers B and C (114 Pa.s^−1^ vs. 36–38 Pa.s^−1^) over the shear-rate range measured, indicating a possible better ability to resist flows and potentially remain in place at the site of application in vivo. The results of storage modulus (G’) measurements showed 100 Pa for polymer A and 16 Pa and 20 Pa for polymers B and C, respectively. This translated into a weaker elastic behavior for gels B and C, and a lower ability to resist sudden deformation. The peel test results showed a rupture strength of 72 mN (0.016 lbf) for polymer A, 39.6 mN (0.0089 lbf) for polymer B, and 38.3 mN (0.0086 lbf) for polymers C, indicating possible higher adhesive properties for polymer A. Tests measuring the extrudability of the hyaluronic acid gels in their commercial syringes showed an average extrusion force of 20 N (4.5 lbf) for polymer A, 28 N (6.33 lbf) for polymer B, and 17 N (3.79 lbf) for polymer C. Conclusions: Modified anti-adhesion gels made of hyaluronic acid differed in mechanical properties and concentration. Further clinical studies are needed to confirm whether these differences make one polymer easier to apply during surgery and more likely to stay in place longer after in vivo application, and to determine which is potentially superior in terms of preventing adhesions.

## 1. Introduction

Adhesions are defined as internal scar tissue that may form as part of the body’s healing process after surgery [1]. They constitute a widespread complication of abdominopelvic surgery, developing in 79–90% of patients who receive open abdominal, pelvic, or hysteroscopy surgery [2,3,4]. Adhesions may cause acute abdominal complications such as bowel obstruction, chronic pelvic pain, female infertility, or some combination of the above, and patients may require reoperation [1,5,6,7,8,9,10]. The formation of the fibrous scar tissue responsible for adhesions is at least partly the result of ischemia and inflammation secondary to perioperative trauma [11], and the critical period in which adhesions appear is the first three to five postoperative days [12].

In the ten years following open surgery, one study documented that approximately one in three patients were readmitted to the hospital for causes possibly related to adhesions, and 5.7% of patients were readmitted for causes directly related to adhesions [13]. Even after the widespread adoption of laparoscopic surgery, and although the incidence of hospital readmissions related to adhesions has been reduced, adhesion-related morbidity remains significant. One in six patients treated laparoscopically are readmitted for a complication possibly related to adhesion, and 1.7% for a complication directly related to adhesion [13].

Although primary prevention based on meticulous surgical techniques to limit ischemic factors and peritoneal inflammation appears to be the best treatment strategy [10], it is not sufficient to prevent postoperative adhesions [2,3,4]. Therefore, the interest in secondary prevention using physical adhesion barriers is growing. The underlying principle here is based on interposing a physical barrier that prevents damaged peritoneal surfaces from making contact until the peritoneum heals [4,5]. At present, such adhesion barriers are rarely used in daily practice, despite existing evidence showing their efficacy in reducing adhesion formation [14,15], and the existence of a large offer of natural and synthetic biomaterials (hyaluronan, alginate, cellulose, starch, polyethylene glycol, etc.) available in different forms (gel, film, sprayable powder, sponge, liquid, etc.) [16,17].

**Figure 1:**
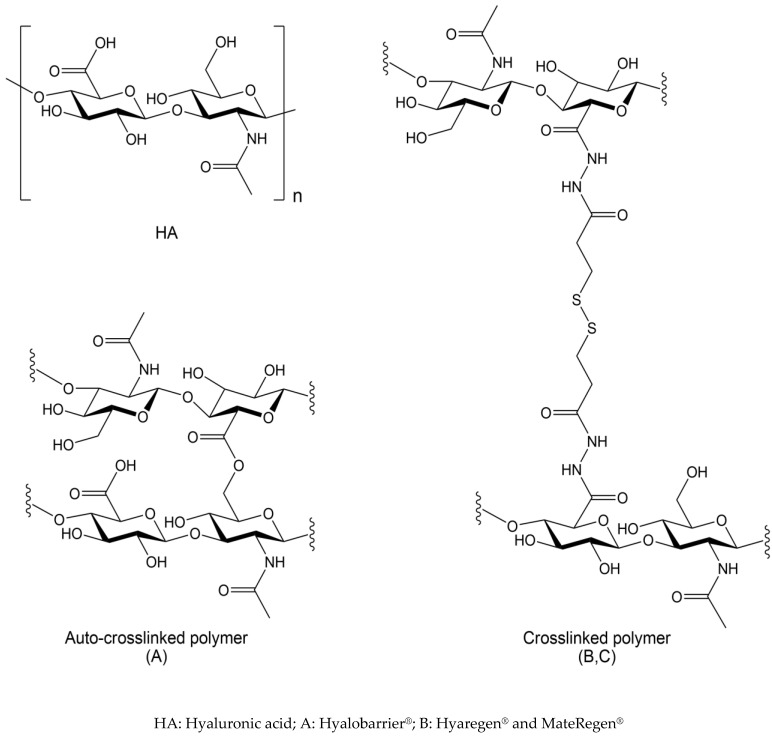
Chemical structure of hyaluronic acid-based polymers.

Hyaluronic acid (HA) is a natural polymer extensively used in biomedical applications due to its excellent biocompatibility, swelling properties, and tunable mechanical properties [18]. HA is a macromolecule of high molecular weight (100,000 to 6 million Daltons) that belongs to the glycosaminoglycan family. Its structure is composed of two sugar-based moieties (N-acetylglucosamine and D-glucuronic acid), which combine to form a repeating disaccharide polymer [19,20] linked via alternating β-1,4 and β-1,3 glycosidic bonds (Figure 1). Derivatives of hyaluronic acid form the basis of a number of anti-adhesion gels [1].

Many physical and chemical properties underlie the physiological and pharmacological characteristics of HA. It is highly hydrophilic, retaining or combining with water to form a tissue-moisturizing complex. From a rheological perspective, HA exhibits viscoelastic and shear thinning properties, thereby cushioning and lubricating skeletal structures. Finally, it has been demonstrated to have antioxidant properties due to its free-radical-scavenging activity [19,20]. HA has been shown to bind to specific receptors on the surface of certain cells involved in cellular adhesion and mobility as well as in inflammatory processes [20,21]. It also interacts with extracellular proteins, binding to collagen during healing and to fibrin during the inflammatory phase [20]. Moreover, HA has the advantage of being of physiological origin, biocompatible, nonallergenic, noninflammatory, and biodegradable [22,23,24]. Hyaluronan is a major component of many body tissues and fluids, where it plays a role in physical support and mechanical protection [25]. Finally, HA has been being utilized to prevent surgical adhesions for over 10 years [26], and its efficacy has been confirmed on several occasions, including recently in a meta-analysis [27]. Different formulations of HA are available for adhesion-prevention therapy.

The aim of this work was to directly compare two types of HA-derived adhesion barriers featuring different reticulation: the established auto-crosslinked Hyalobarrier^®^ gel (Anika Therapeutics) with a zero-length crosslinking, and the more recent crosslinked HyaRegen^®^ and MateRegen^®^ gels (BioRegen) with a chemically modified longer crosslink. In this laboratory test we evaluate how these two types of polymers differ in their chemistry, formulation, and mechanical properties.

## 2. Materials and Methods

The analysis was conducted under laboratory-controlled conditions on the HA-based products described in Table 1. The following aspects were investigated: chemistry, formulation, and mechanical properties.

The flow properties of the different compounds were analyzed, assessing steady-shear viscosity and storage modulus using a TA Instruments rheometers, model DHR-1 and AR-1500ex. We employed the a cone–plate geometry for the analysis (hard anodized aluminum, ST, 40 m, 2°, Smart Swap, TA Instruments, New Castle, DE, USA). To determine steady-shear viscosity, a rotational rheometer, TA Instruments AR-1500ex, with a 60 mm, 1° cone at 20 °C, was used. The viscosity response was observed at a shear rate of 1.0 s^−1^. For storage modulus (G’), that is, the elastic component, the small-angle oscillatory shear test was used, measuring the elasticity over a range of frequencies. The more rapidly the material is deformed, the stronger the elastic effects are, and the higher the frequency, the more rapid the deformation. Typically, G’ increases with frequency.

A peel test was conducted on both types of anti-adhesion gel to determine their adhesive strength to a hydrophilic surface, simulating human tissue. The hydrophilic surface was created by wetting paper strips. The gels were applied to the paper using a glass mold to give a uniform coating of 21 mm × 75 mm × 1 mm in size. The piece of paper was applied to the top of the gel. The sample was then placed in a tensile tester and the force measured as the strips were pulled apart at a rate of 330 mm/min until the materials separated, corresponding to the rupture strength. Experiments were performed at room temperature on a MARK-10 instrument, Motorized Test Stand ESM301L model (Mark-10 Corporation, Copiague, NY, USA)

Extrusion force tests were performed on both types of gels to determine their extrudability (i.e., the force required to push the gel out of the cannula), using the same MARK-10 instrument with the force sensor traveling downwards in a compression mode. Extrusion force tests were performed on all three gels using a 5 cm cannula with an inside diameter of 2.0 mm, at a linear rate of 12 mm/min, at room temperature. During the test, the gel was compressed until it flowed out of the cannula, corresponding to the extrusion force. In this case, the gels were not transferred into the same-sized syringe, but rather the test was performed using syringes from the original product. Thus, the extrusion force comparison reflected the actual usage.

## 3. Results

### 3.1. Chemical Structure

The HA-based products are described in Table 1, and their chemical structures are depicted in Figure 1. In the auto-crosslinked polymer A, the HA chains are linked by an ester function, formed by a condensation reaction (displacement of a water molecule) between the free primary hydroxyl function and the carboxylic acid residue of the HA chains. This is often referred to as a ‘zero-length’ crosslink, as no foreign moiety is introduced into the material. The final product is a reticulated form of HA in a gel form that has only been chemically modified to a minimal degree. The hydrolysis of the ester functional groups reversibly regenerates unmodified HA.

The crosslinked polymer (products B and C) is a chemically modified, reticulated polymer of HA, crosslinked by a long chain between the carboxylic acid functional groups of the HA chains, containing two hydrazide functional groups and a disulfide bridge in the center (Figure 1).

### 3.2. Formulation and Material Properties

The main evaluated physical properties of both types of polymers are summarized in Table 2. According to the manufacturer specifications, the two types of gels have different HA concentrations, with gel A being approximately six times more concentrated than gel B (30 mg/mL vs. 5 mg/mL). To demonstrate the ability of the polymers to resist flow and remain in place at the site of application, steady-shear viscosity tests were performed on each gel. The test results in the comparative flow curves show that the steady-shear viscosity of all polymers decreased with flow rate, demonstrating viscoelastic behavior. The results also show that polymer A had a shear viscosity approximately three times as large as polymers B and C (114 Pa.s^−1^ vs. 36–38 Pa.s^−1^) over the shear-rate range measured. These differences in physical properties could be attributable to the different synthesis methods of the polymers being evaluated. 

**Figure 2:**
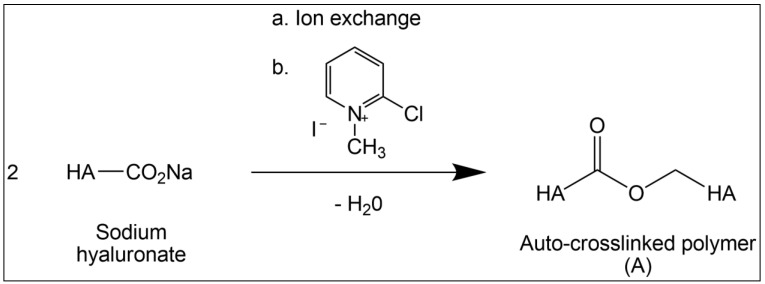
Synthesis of hyaluronic acid auto-crosslinked polymer gel (Hyalobarrier^®^).

The formation of polymer A is depicted in Figure 2. The *t*-butyl-ammonium hyaluronate intermediate is formed from sodium hyaluronate via an ion-exchange step. This hydrophobic salt of HA is then dissolved in N-methyl-pyrrolidone (NMP), where the 2-chloro-1-methyl pyridinium iodide reagent is introduced to react with the carboxylic group of the glucuronic acid unit, forming an ‘active’ ester. The ester is displaced by the primary hydroxyl group on C-6 of N-acetylglucosamine of another chain or another part of the same chain, forming the ‘zero-length’ ester bond. The level of impurities in the resulting auto-crosslinked gel is controlled. The purity was assessed using two orthogonal methods, a colorimetric analysis by spectrometry and a chromatographic analysis by gel-permeation chromatographic HPLC. The presence of any potential process impurities was quantified to be less than 0.2% *w*/*w*.

**Figure 3:**
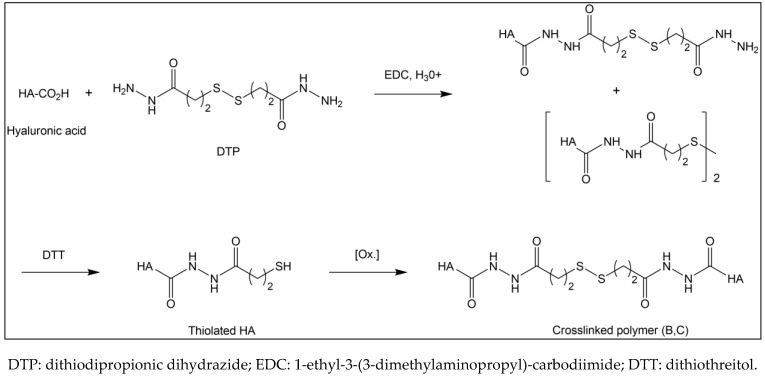
Synthetic route of hyaluronic acid crosslinked polymers B and C (HyaRegen^®^ and MateRegen^®^).

Condensation reaction (displacement of a water molecule) yields an ester link between the HA chains. The crosslinked polymers B and C are chemically identical and produced according to the synthetic route shown in Figure 3. They are synthesized by chemically modified HA chains, linking the carboxylic acid groups of glucuronic acid moieties with hydrazides as a cross-link [28,29,30,31].

The process is as follows: HA is activated with 1-ethyl-3-(3-dimethylaminopropyl)-carbodiimide (EDC) under acidic conditions in aqueous media, forming an O-acylisourea side group on the HA chain. Then, dithiodipropionic dihydrazide (DTP) reacts with this modified HA, displacing the O-acylisourea and forming amides or hydrazides, which completes the reaction. The disulfide bond is reduced by dithiothreitol (DTT) to thiol groups, breaking the crosslink and generating thiolated HA. The solution is then clarified by centrifugation and the purity of the thiolated HA measured by gel-permeation chromatography (GPC) and ^1^H NMR. The thiolated material is subsequently loaded into syringes and whatever dissolved oxygen is present oxidizes some percentage of the thiol groups back to a disulfide bond, forming the final gel (Figure 3). The final purity level required for crosslinked gels B and C is not known, nor is any toxicological information available for either the dithiodipropionic acid esters (reaction intermediate) or the dithiodipropionic dihydrazide reagent.

**Figure 4:**
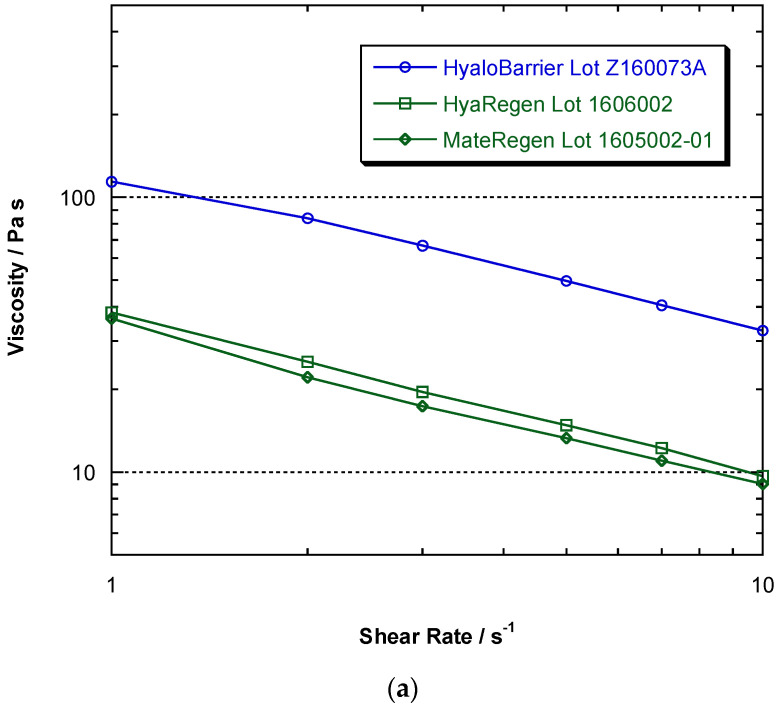

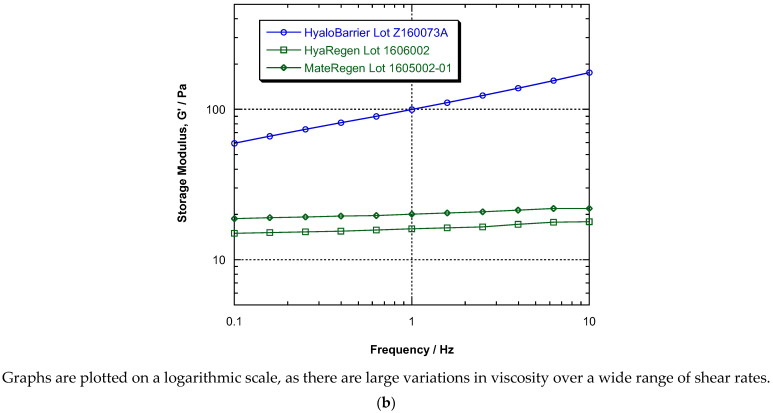
Comparison of physical characteristics of hyaluronic acid gels. (**a**) Comparison of viscosity vs. shear rate flow curves for gel A (Hyalobarrier^®^) and gels B and C (HyaRegen^®^ and MateRegen^®^). (**b**) Comparison of storage moduli G’ versus frequency for hyaluronic acid-based products.

The flow properties of modified HA gels were studied to demonstrate viscoelastic behavior by measuring steady-shear viscosity over a large range of shear rates. The results in Figure 4 show that all gels exhibited this behavior, highlighted by the flow curves that decrease with shear rate. It can also be observed that Hyalobarrier^®^ had a shear viscosity approximately three times higher than HyaRegen^®^ and MateRegen^®^ throughout the range of shear rates studied.

To evaluate the elastic component, also called the storage modulus or G’, we compared all polymers by measuring G’ over a range of frequencies. The results (Figure 4a) showed that the dependence of G’ on frequency for products B and C was weak, and these polymers acted more like simple elastic solids. Hence, their resistance to a sudden deformation did not increase as it did for product A, which was higher at all measured frequencies. At low frequency (0.1 Hz), the resistance of product A was about four times greater, and at the higher frequency of 10 Hz, G’ was about ten times greater (Figure 4b).

**Figure 5:**
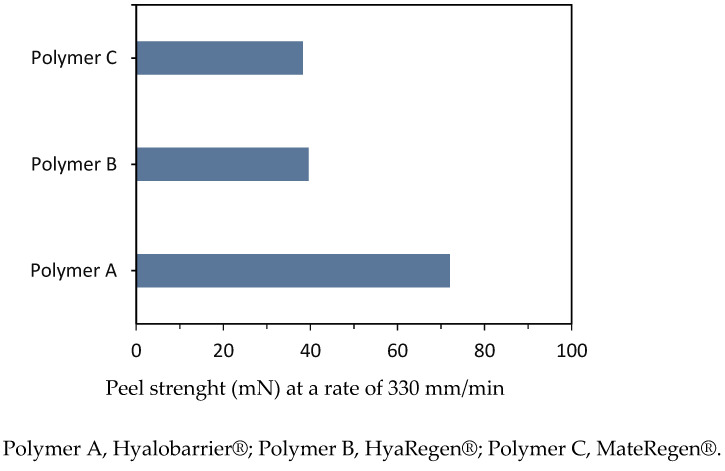
Peel strength comparison between hyaluronic acid gels.

To compare the adhesive strength between hyaluronan polymers, peel tests were conducted on all three gels under the same experimental conditions at a rate of 330 mm/min. The average forces measured are presented in Table 2 and illustrated in Figure 5. The results indicate that the rupture strength of polymer A (72.0 mN) was approximately two times higher than the rupture strengths of polymers B and C (39.6 and 38.3 mN, respectively).

**Figure 6:**
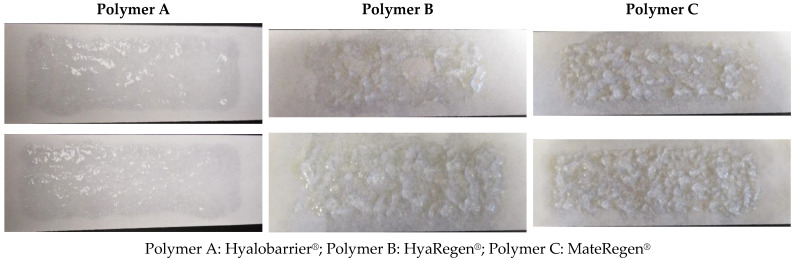
Photographs of peel strips after completing two peel strength tests on all three polymers.

Peel tests also revealed an interesting qualitative result. The photographs of the two peel strips after completing the peel strength test shown in Figure 6 indicate that polymer A adhered strongly to the strips and that the fracture occurred in the bulk of the material. There was a relatively even coating on both strips (Figure 6—Polymer A).

In the case of polymer B, peel strip photographs show large clumps and some bare patches without gel, indicating a weaker adherence to the hydrophilic paper surface (Figure 6—Polymer B). This indicates that the inherent gel particle size of gel B was larger and that the coating was not as uniform as that of gel A.

No difference was observed between the peel strength test results of polymers B and C (Figure 6).

**Figure 7:**
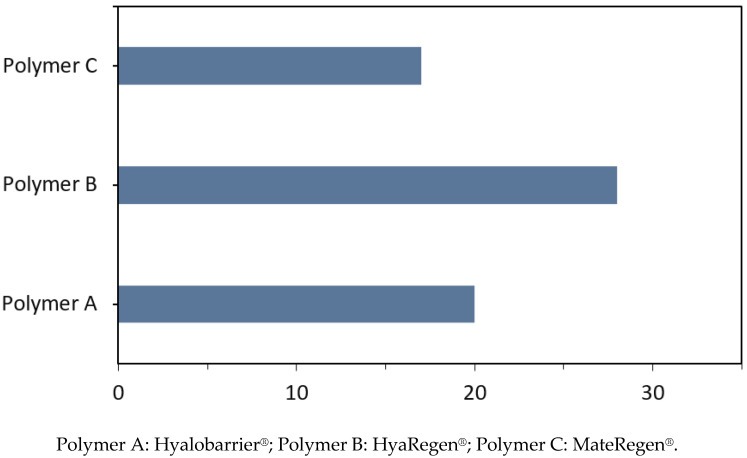
Comparison of average extrusion forces between HA gels in their commercial syringes.

The average extrusion forces were measured for all three polymers in their commercial syringes to better reflect actual product use. The average forces measured are presented in Table 2 and illustrated in Figure 7.

For polymer A, the measured force was 4.5 lbf (20 N). For polymer B, an extrusion force of 6.33 lbf (28 N) was observed, and a force of 3.79 lbf (17 N) was observed for polymer C. Due to the larger syringe diameter of gel B, it was expected to lead to an extrusion force greater than 6 lbf (27 N) (Table 2). The extrusion force on the 5 mL syringe of gel C was expected to be lower, given the low steady-shear viscosity observed for this crosslinked polymer, and this was borne out by the results.

## 4. Discussion

Despite the well-known effects of careful surgical technique in preventing the formation of adhesions [31], the burden of disease caused by postsurgical adhesions [13] has pushed the industry to produce safe and effective agents to minimize the damage that surgical trauma induces in peritoneal surfaces and organs [32,33]. Different products based on several chemically modified substances are now available. HA is currently one of the main compounds used in new products because it has anti-inflammatory properties and characteristics that play important roles in the wound healing process [34,35]. Furthermore, HA itself can serve as a barrier between tissues. Nevertheless, there are differences between products containing HA.

The anti-adhesive efficacy of the two types of HA-based products evaluated herein following gynecologic laparoscopic surgery has not yet been directly compared, although they were both demonstrated to be efficacious in prospective, randomized, placebo-controlled, observer-blinded studies [36,37,38,39,40]. Our analysis is the first one designed to determine the differences between these HA polymers with regard to their chemical structure and material properties in crosslinked (HyaRegen^®^/MateRegen^®^) and auto-crosslinked (Hyalobarrier^®^) anti-adhesion agents.

Regarding their chemical structure, both types of gel contain modified reticulated forms of HA. However, we observed a difference in the auto-crosslinked structure of polymer A, with no foreign moiety being introduced into the material. Under physiological conditions, gel A degrades by hydrolysis (addition of a water molecule), reforming the original, unmodified HA, and the polysaccharide backbone breaks down into carbon dioxide and water. Meanwhile, the HA chains of the crosslinked gels (B and C) are crosslinked by dihydrazide groups, with a new disulfide bridge in the center of the link. This is the result of hydrazine being used during synthesis. The hydrazide bond is quite stable, and it is likely that it remains intact and degrades only in vivo, while the HA chains themselves are substantially degraded. Further studies should explore foreign-body reactions during clinical use based on the structural differences of the two products. Whether the residual presence of hydrazine after the degradation of gels B and C could induce changes in hepatic function, as reported after prolonged high-dose exposure (8 h time-weighted average permissible exposure limit of 1 ppm or 1.3 mg/m^3^), should also be evaluated [41].

On the other hand, the flow properties of HA-based gels, and specially modified HA solutions, are complex. These systems exhibit viscoelastic behavior, often with a critical yield stress. These fluids are also ‘shear-thinning’ or pseudoplastic. The resistance to flow does not increase linearly with the flow rate: twice the flow rate requires less than twice the force. As viscosity represents the ratio of the driving force to the flow rate, steady-shear viscosity decreases with increasing flow rate. Gel A showed a shear viscosity approximately three times higher than those of gels B and C over the range of measured shear rates. This indicates that gel A is ‘thicker’, and therefore more likely to resist flow and remain in place at the application site in vivo.

Regarding the materials’ ability to store energy elastically, all gels were demonstrated to be viscoelastic. Elastic effects are usually measured by small-angle oscillatory shear tests, which characterize the ability of a material to deform rapidly in response to oscillatory shear applied at different frequencies. The higher the frequency, the faster the deformation, and the stronger the elastic effect. the measured dependence of G’ on frequency for products B and C was weaker. Their resistance to a sudden deformation did not increase with frequency as it did for product A. Indeed, the elastic modulus of gel A was higher at all measured frequencies, making this substance more likely to stay in place after application.

The rupture strength of polymer A was observed to be twice as high as polymers B and C, and photographs showed a weaker adherence to the hydrophilic paper surface for gels B and C, with larger gel particle sizes and less-uniform coatings.

It was not possible to draw preliminary conclusions about the high extrusion force measured on gel C with the 5 mL syringe. This requires further investigation of the yield stress properties of this polymer.

## 5. Conclusions

Auto-crosslinked polymer A and crosslinked polymers B and C are chemically modified HA gels used to prevent adhesions from developing after surgery. This first comparative study showed several chemical and mechanical differences between these types of polymers.

They differ in their chemical structure. Polymer A has a short, or ‘zero length’ cross-linking, resulting from a simple displacement of water molecules, with no foreign materials. Polymers B and C, on the other hand, present a longer cross-linking incorporating foreign fragments coming from the use of other chemical reagents during their synthesis, for which total hepatic innocuity remains to be demonstrated. They also differ in their concentration and mechanical properties, with gel A having a higher concentration of hyaluronic acid, a higher rupture strength, and higher viscoelastic properties than gels B and C.

While these initial results indicate that polymer A is more likely to resist flows and peeling than polymers B and C, further clinical studies directly comparing these gels are needed to confirm whether their physicochemical differences make one polymer easier to apply during surgery and more likely to stay in place longer after application in vivo, and to determine which is potentially superior in terms of adhesion prevention.

## Figures and Tables

**Table 1:** Description of the products.

	Auto-Crosslinked Polymer A	Crosslinked Polymers	
		B	C
	Hyalobarrier^®^	HyaRegen^®^	MateRegen^®^
Syringe filling (mL)	10	10/20	5
Claimed HA concentration (mg/mL)	30	5	N/A
Shelf life	3 years	2 years	2 years
Storage conditions	2–8 °C	2–30 °C	2–30 °C

N/A: not available.

**Table 2:** Summary of the evaluated physical characteristics of the products.

	Polymers		
	A	B	C
Shear viscosity (Pa.s^−1^)	114	38	36
Storage modulus G’ (Pa)	100	16	20
Peel strength	0.016 lbf 72 mN	0.0089 lbf 39.6 mN	0.0086 lbf 38.3 mN
Extrusion force	4.5 lbf 20 N	6.33 lbf 28 N	3.79 lbf 17 N

lbf: pound-force; mN: milli-Newton; N: Newton; Pa: Pascal; Pa.s^−1^: Pascal-seconds.

## Data Availability

Not applicable.
